# A defect in Δ^6 ^and Δ^5 ^desaturases may be a factor in the initiation and progression of insulin resistance, the metabolic syndrome and ischemic heart disease in South Asians

**DOI:** 10.1186/1476-511X-9-130

**Published:** 2010-11-09

**Authors:** Undurti N Das

**Affiliations:** 1 UND Life Sciences, 13800 Fairhill Road, #321, Shaker Heights, OH 44120, USA; 2 School of Biotechnology, Jawaharlal Nehru Technological University, Kakinada-533 003, India

## Abstract

The high incidence of insulin resistance and the metabolic syndrome in South Asians remains unexplained. I propose that a defect in the activity of Δ^6 ^and Δ^5 ^desaturases and consequent low plasma and tissue concentrations of polyunsaturated fatty acids such as γ-linolenic acid (GLA), dihomo-γ-linolenic acid (DGLA), arachidonic acid (AA), eicosapentaenoic acid (EPA) and docosahexaenoic acid (DHA) and formation of their anti-inflammatory products prostaglandin E_1 _(PGE_1_), prostacyclin (PGI_2_), PGI_3_, lipoxins, resolvins, protectins, maresins and nitrolipids could be responsible for the high incidence of insulin resistance, the metabolic syndrome and ischemic heart disease (IHD) in South Asians. This proposal is supported by the observation that South Asian Indians have lower plasma and tissue concentrations of GLA, DGLA, AA, EPA and DHA, the precursors of PGE_1_, PGI_2_, PGI_3_, lipoxins, resolvins, protectins, and nitrolipids, the endogenous molecules that prevent platelet aggregation, vasoconstriction, thrombus formation, leukocyte activation and possess anti-inflammatory action and thus, are capable of preventing the development of insulin resistance, atherosclerosis, hypertension, type 2 diabetes mellitus and premature ischemic heart disease. Genetic predisposition, high carbohydrate intake, lack of exercise, tobacco use and low birth weight due to maternal malnutrition suppress the activity of Δ^6 ^and Δ^5 ^desaturases that leads to low plasma and tissue concentrations of polyunsaturated fatty acids and their products. This implies that adequate provision of polyunsaturated fatty acids and co-factors needed for their metabolism, and efforts to enhance the formation of their beneficial metabolites PGE_1_, PGI_2_, PGI_3_, lipoxins, resolvins, protectins, maresins and nitrolipids could form a novel approach in the prevention and management of these diseases in this high-risk population.

## Introduction

The high prevalence of ischemic heart disease in South Asians has been attributed to abdominal obesity, tobacco use, dyslipidemia, hypertension, diabetes mellitus, abdominal obesity and lack of exercise [1]. All these factors are known to be associated with insulin resistance that could account for the high incidence of ischemic heart disease in South Asians [2]. Almost 60% of the world's heart disease is expected to occur in South Asians. Hence, it is essential that pathophysiology of the disease as to why it is common in South Asians needs to be understood to develop relevant preventive and therapeutic strategies.

In a study that evaluated the differences in postprandial glycemia and insulin sensitivity among young adults of different ethnic origins, it was noted that young South East Asians had the highest postprandial glycemia and lowest insulin sensitivity, whereas European and Arabic Caucasian subjects were the most insulin sensitive and carbohydrate tolerant. These findings suggest that insulin resistance is evident even in lean, young adults of South Asian origin even when they are healthy [3]. In addition, ethnicity appears to be an important risk factor for type 2 diabetes in dysglycaemic persons. In a 2-by-2 factorial double-blind randomized controlled trial that compared the effects of rosiglitazone and ramipril on the primary outcome of diabetes or death in persons meeting criteria for impaired glucose tolerance or impaired fasting glucose, it was noted that South Asians experienced a smaller, and Latinos a larger preventive effect [4], suggesting that South Asians respond less favorably to the preventive effects of rosiglitazone. But, none of these studies could pinpoint the underlying cause for the higher incidence of insulin resistance in South Asians.

## Polyunsaturated fatty acids enhance cell membrane fluidity and decrease Insulin resistance

Insulin resistance is common in obesity, type 2 diabetes mellitus, essential hypertension, hyperlipidemia, ischemic (coronary) heart disease, atherosclerosis, ageing, polycystic ovarian disease and the metabolic syndrome. Increased consumption of energy dense food induces insulin resistance, leads to the development of obesity, the precursor of the metabolic syndrome. Calorie restriction, reduced food intake, increase in energy expenditure in the form of exercise attenuates insulin resistance, decreases the incidence of obesity, type 2 diabetes mellitus, hypertension and the metabolic syndrome.

Insulin has to bind to its receptors on the cell membrane to bring about its actions. Hence, cell membrane structure and its functional integrity influence the properties of the insulin receptor including its affinity to insulin. The properties of cell membrane especially, its fluidity depend on the lipid constitution of the membrane. High saturated fatty acid(s) content of the membrane will render it more rigid (i.e. decrease in fluidity) that leads to a decrease in the number of insulin receptors and the affinity of insulin to its receptors [5-11]. This, in turn, causes insulin resistance and associated hyperinsulinemia. It was reported that an increase in the amount of polyunsaturated fatty acids in the cell membrane may produce a significant increase of the number of high-affinity sites with a concomitant decrease of low-affinity sites for insulin [9,11].

These in vitro results are supported by in vivo experiments wherein it was observed that feeding animals with diets high in saturated fat induced insulin resistance, and replacing saturated fat isocalorically with polyunsaturated fat, especially long-chain ω-3 fatty acids, prevented the development of insulin resistance in skeletal-muscle tissue. The content of fatty acids in sarcolemmal phospholipid was significantly related to the dietary composition. Insulin binding to intact sarcolemmal vesicles prepared from rats fed on diets high in ω-3 fatty acids increased 14-fold compared with animals fed on the low-ω-3 diet and the former showed increased sarcolemmal insulin binding by 2.3-fold. Increased insulin binding was due to increased receptor number at the low-affinity high-capacity binding site, suggesting that dietary ω-3 and polyunsaturated fatty acids increase insulin binding to sarcolemma by changing the fatty acyl composition of phospholipid surrounding the insulin receptor, an action by which dietary fatty acids seem to modify insulin action [12].

In addition, diet rich in fish oil, a rich source of EPA and DHA, prevented obesity, hyperlipidemia and adipocyte insulin resistance in rats [13,14] lending support to this view.

## Mechanisms of anti-atherosclerotic actions of PUFAs

Although the exact mechanism by which PUFAs/EFAs decrease insulin resistance is not known, some of the possibilities include: (a) increase in the number of insulin receptors due to increased membrane fluidity; (b) an increase in GLUT-4 mRNA and protein level in adipocytes [15]; (c) formation of anti-inflammatory and anti-atherosclerotic molecules such as PGI_2_, PGE_1_, lipoxins, resolvins, protectins and maresins [16-21]; (d) suppression of the expression of adhesion molecules [22-24]; (e) inhibition of the formation of pro-inflammatory cytokines tumor necrosis factor-α (TNF-α), macrophage migration factor (MIF), high-mobility group box 1 (HMGB1) and interleukin-6 (IL-6) [25-27]; (f) enhancement in the formation of brain-derived neurotrophic factor (BDNF) in the brain and gut that has anti-diabetic actions [28-30]; and that (BDNF), in turn, augments PGI_2 _synthesis [31], a potent platelet anti-aggregator, vasodilator and anti-atherosclerotic molecule; (f) enhancement in the synthesis and action of BMPs (bone morphogenetic proteins) that enhance the growth and development of the brain [32,33]; (g) modulation of the growth and actions of gut bacteria [34-37]; (h) binding to various nuclear receptors and correction of dyslipidemia [38-42]; and (i) regulation of both the secretion and actions of various hypothalamic neurotransmitters and peptides that regulate appetite, satiety, food intake and insulin secretion [43-49]. Furthermore, insulin enhances the activity of Δ^6 ^and Δ^5 ^desaturases [50] and thus, potentially augments the formation of PUFAs that, in turn, enhance the action of insulin. It is likely that when the intake of PUFAs is adequate (it should be adequate throughout life, especially during the formative periods of various cells and tissues and programming of their functions and responses to various endogenous and exogenous stimuli and stresses including perinatal period), the incorporation of these fatty acids into the cell membrane of endothelial cells will be optimum such that the production of PGI_2_, PGE_1_, lipoxins, resolvins, protectins, maresins and nitrolipids will be sufficient both during health and in instances of exposure to adverse stimuli such as shear stress so that the expression of adhesion molecules and the production of pro-inflammatory cytokines will be suppressed to prevent platelet aggregation and initiation and progression of atherosclerosis. Thus, for the maintenance of optimal health of endothelial cells adequate intake of PUFAs though out life both during the perinatal period and adult life is necessary. In the absence of adequate amounts of PUFAs, endothelial cells will not be able to synthesize and release sufficient quantities of PGE_1_, PGI_2_, PGI_3_, lipoxins, resolvins, protectins, maresins and nitrolipids that could lead to endothelial dysfunction, insulin resistance and finally the initiation and progression of the metabolic syndrome and ischemic heart disease.

## South Asians are deficient in beneficial PUFAs

**Figure 1:**
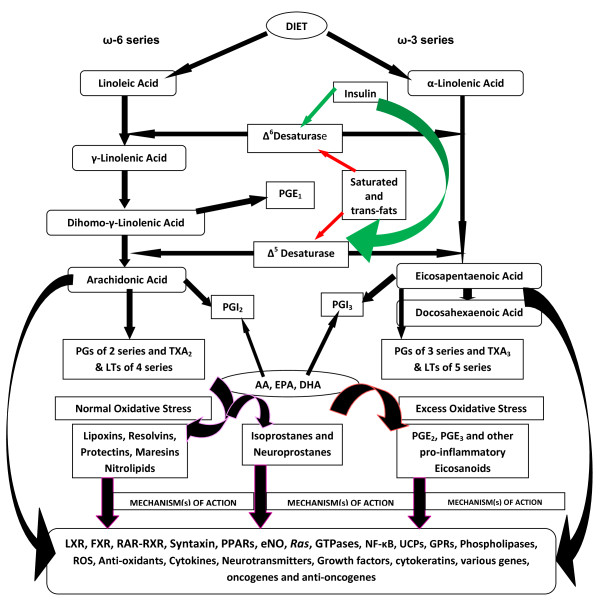
**Metabolism of essential fatty acids (EFAs)**. Red arrows indicate inhibition of activity of Δ^6 ^and Δ^5 ^desaturases while green arrows indicate enhancement of their activity. It is predicted that under normal oxidative conditions adequate formation of PGE_1_, PGI_2_, PGI_3_, lipoxins, resolvins, protectins, maresins and nitrolipids will occur that would prevent low-grade systemic inflammation and insulin resistance. Isoprostanes and neuroprostanes are also formed from PUFAs under certain specific circumstances that have anti-inflammatory and neuroprotective properties. On the other hand, under conditions of excess oxidative stress not only the formation of PGE_1_, PGI_2_, PGI_3_, lipoxins, resolvins, protectins, maresins, nitrolipids and endothelial nitric oxide is decreased or abrogated but would also lead to the formation of excess of pro-inflammatory eicosanoids such as PGE_2_, PGE_3_, PGF_2α_, PGF_3α_, leukotrienes and thromboxanes. PUFAs, various eicosanoids, lipoxins, resolvins, protectins, maresins, nitrolipids and endothelial nitric oxide may bring about their various actions by acting or modulating nuclear receptors, LXR, FXR, RAR-RXR, Syntaxin, PPARs, eNO, *Ras*, GTPases, NF-κB, UCPs, G-protein coupled receptors (GPRs),phospholipases, ROS, anti-oxidants, cytokines, neurotransmitters, growth factors, cytokeratins, various genes, oncogenes and anti-oncogenes.

If this proposal is true, it implies that South Asians are deficient in PUFAs. Previously, I showed that even healthy Indians have lower concentrations of various PUFAs compared to Canadians and Americans (USA) [51], suggesting that Indians have low activity of Δ^6 ^and Δ^5 ^desaturases (see Figure 1 for metabolism of essential fatty acids). This, in turn, could lead to decreased formation of PGE_1_, PGI_2_, PGI_3_, lipoxins, resolvins, protectins, maresins and nitrolipids due to precursor PUFA deficiency. This is further supported by the observation that South Asians with IHD have much lower levels of PUFAs in their plasma phospholipid fraction compared to healthy subjects [52]. Furthermore, low activity of Δ^6 ^and Δ^5 ^desaturases could lead to the initiation and progression of insulin resistance and atherosclerosis [53,54]. These evidences suggest that South Asians are at high risk of developing type 2 diabetes mellitus, the metabolic syndrome, IHD and its associated death and complications due to a defect in the activity of Δ^6 ^and Δ^5 ^desaturases that result in low plasma and tissue concentrations of PUFAs and their products: PGE_1_, PGI_2_, PGI_3_, lipoxins, resolvins, protectins, maresins and nitrolipids that are necessary to prevent platelet aggregation, lower blood pressure, reduce LDL-C, ameliorate the adverse actions of homocysteine, and inhibit ACE (angiotensin converting enzyme) and HMG-CoA enzyme activities [20,55]. In addition, PUFAs and their beneficial products such as PGI_2 _and lipoxins have anti-arrhythmic action, enhance wound healing and suppress inflammation [19-21,56-58].

Folic acid that reduces plasma homocysteine levels augments the metabolism of essential fatty acids such that plasma and tissue levels of various PUFAs are enhanced [59,60]. Similar potentiating action of vitamin B_6 _on Δ^6 ^and Δ^5 ^desaturases was also reported [59-62]. Recently it was noted that the gene that encodes the 5-lipoxygenase activating protein (FLAP) and its risk variant results in an almost 2-fold increased risk of IHD by leading to the production of leukotriene B_4 _(LTB_4_), a potent chemokine mediators of arterial inflammation [63,64]. This is supported by the observation that patients with IHD produce more LTB_4 _than controls, suggesting that LTB pathway is upregulated in IHD. It is interesting to note that lipoxins are potent inhibitors of LTB_4 _and leukotrienes [65,66].

These evidences suggest that South Asians are at high risk to develop insulin resistance, type 2 diabetes mellitus, the metabolic syndrome and IHD due to low levels of various PUFAs, LXs, resolvins, protectins, maresins and nitrolipids, and hence could benefit from supplementation of PUFAs. Furthermore, South Asians consume less amounts of ω-3 PUFAs and more of saturated and trans-fats that are known to inhibit the activity of Δ^6 ^and Δ^5 ^desaturases [6,7,16-18] which, in turn, further aggravate their tendency to develop insulin resistance and the metabolic syndrome [67]. Since, South Asians have low plasma levels of various PUFAs and consume significantly lower quantities of ω-3 PUFAs; they are more prone to have low-grade systemic inflammation. This is supported by the observation that of 1250 adults of South Asian, Chinese, European and Aboriginal ancestry studied; the age- and sex-adjusted mean CRP was 3.74 ± 0.14 mg/L among Aboriginals, 2.59 mg/L ± 0.12 among South Asians, and 1.18 ± 0.13 mg/L among Chinese compared with 2.06 ± 0.12 mg/L among Europeans (overall P < 0.0001). Differences in the CRP concentration between ethnic groups were substantially diminished, but not abolished, after adjustment for metabolic factors. CRP was independently associated with CVD (cardiovascular diseases) after adjusting for the Framingham risk factors, atherosclerosis, anthropometric measurements, and ethnicity (OR = 1.03 for a 0.1-increase in CRP; P = 0.02). These results clearly indicate that South Asians are more prone to have low-grade systemic inflammation compared to other ethnic groups [68] that may render them highly susceptible to cardiovascular diseases.

## Leukocyte activation and atherosclerosis

One of the major health issues in South Asians is the occurrence of premature atheroslcerosis and consequent IHD that, in turn, is due to insulin resistance. Leukocytes seem to play a major role in the pathobiology of atherosclerosis. Thus, peripheral leukocytes could be used as a marker of insulin resistance and atheroslcerosis by studying the metabolism of essential fatty acids, eicosanoids, lipoxins, resolvins, protectins, maresins, nitrolipids and nitric oxide in these cells. Since leukocytosis is also a marker of inflammation, study of leukocytes and their functions could reflect the underlying pathobiology of low-grade systemic inflammation and its consequences. For instance, higher leukocyte count is associated with a greater cardiovascular risk [69]. This implies that leukocyte myeloperoxidase (MPO) could serve as a biomarker of cardiovascular diseases as reported by Morrow et al [70].

Infiltration of intima by leukocytes and macrophages is an early event to occur in atherosclerosis, suggesting that leukocyte activation is an early event in atherosclerosis. Since atherosclerotic lesions occur in a patchy manner and develop preferentially at bifurcations, branch points, and inner curvatures of arteries, it suggests that local factors play a significant role in the development of atherosclerosis. Hemodynamic forces induce the expression of pro-inflammatory genes [71] that initiate and accelerate atherosclerosis at these points of shear stress. Normocholesterolemic C57BL/6 mice and rabbits showed activation of NF-κB and elevated expression of VCAM-1 and ICAM-1, upregulation of pro-inflammatory genes IL-1, IL-6, MCP-1, as well as antioxidant genes glutathione peroxidase and glutathione-S- transferase 2 in endothelial cells in atherosclerosis-susceptible regions of the ascending aorta [53,71]. Intimal accumulation of LDL and its oxidation products preceded monocyte recruitment into early atherosclerotic lesions, suggesting that lipid accumulation triggers inflammatory response characterized by upregulation of the expression of chemokines and adhesion molecules in the lesion-prone areas in the intima that contributes to leukocyte accumulation and atherosclerotic lesion formation [72-75]. Thus at atheroslcerosis-prone regions of the normal intima, inflammatory response is triggered in response to risk factors that up regulate several proinflammatory genes, which mediate accumulation of leukocytes and initiation and perpetuation of atherosclerosis.

Healthy endothelial cells have the ability to prevent excess expression of adhesion molecules, resist increases in LDL and cholesterol transport and retention, abrogate activation of NF-κB and the induction of expression of pro-inflammatory genes induced by hemodynamic forces at atherosclerosis prone regions due to enhanced infiltration by monocytes, CD68^+ ^leukocytes, and macrophages by elaborating factors/molecules that counter pro-atherosclerotic events. The patchy nature of atherosclerosis suggests that arterial walls undergo regional disturbances of metabolism that include the uncoupling of respiration and oxidative phosphorylation, which may be characteristic of blood vessels being predisposed to the development of atherosclerosis [76]. Oxidative stress, and abnormalities of uncoupling proteins produce smooth muscle contraction and cause hypertension [77], and respiratory uncoupling is increased in the aortae of experimental animals that are susceptible to atherosclerosis [76]. Bernal-Mizrachi *et al *[78] showed that UCP-1 expression in aortic smooth muscle cells causes hypertension and increases atherosclerosis without affecting cholesterol levels. This increase in UCP-1 expression enhanced superoxide anion production and decreased the availability of nitric oxide (NO), suggesting that oxidative stress has been elevated. Thus, inefficient metabolism in blood vessels causes atherosclerosis.

One of the earliest signs of atherosclerosis is the development of abnormal mitochondria in smooth muscle cells [79]. Arteries have marginal oxygenation [80] and hypoxia reduces the respiratory control ratio. Uncoupled respiration precedes atherosclerosis at lesion-prone sites but not at the sites that are resistant to atherosclerosis [76]. Disease-free aortae have abundant concentrations of the essential fatty acid (EFA)-linoleate and possibly anti-inflammatory lipoxins, resolvins, protectins, maresins and nitrolipids whereas fatty streaks are deficient in EFAs [78,81,82]. EFA deficiency promotes respiratory uncoupling [83,84] and atherosclerosis [53,78,85]. Hence, local disturbances of metabolism in the arterial wall are responsible for atherosclerosis and vascular diseases such as IHD.

Aspirin converts arachidonic acid (AA; 20:4 ω-6), eicosapentaenoic acid (EPA; 20:5 ω-3) and docosahexaenoic acid (DHA; 22:6 ω-3) to form aspirin-triggered 15 epimer LXs (ATLs) by activated leukocytes that inhibit acute inflammation (reviewed in 16-18). These 15-epimeric LXs prevent local inflammation on the vessel wall by regulating the motility of PMNs, eosinophils, and monocytes. LXs deficiency leads to an interaction between PMN and endothelial cells that result in endothelial damage, initiation and progression of atherosclerosis [86]. LXs, resolvins and protectins inhibited cytokine generation, leukocyte recruitment, leukocyte diapedesis, exudate formation, suppress the production of pro-inflammatory cytokines. Hence, local deficiency of LXs, resolvins and NPD1 could initiate atheroslcerosis. Furthermore, lipoxins suppress production of MPO from activated leukocytes [87]. Increased generation of MPO by leukocytes could be an indication of decreased formation of lipoxins, resolvins, protectins, maresins and nitrolipids by endothelial cells. This implies that enhancing the formation of endothelial LXs, resolvins, protectins, maresins and nitrolipids may suppress leukocyte activation and MPO generation and prevent IHD.

In this context, it is interesting to note that elevated circulating platelet-monocyte complexes correlated significantly with elevated aortic carotid-femoral pulse wave velocity and platelet-monocyte complexes were higher in subjects with femoral plaques of South Asian descent and Europeans. Higher numbers of platelet-monocyte complexes were independently related to elevated levels of C-reactive protein (CRP), higher carotid-femoral pulse wave velocity, hypertension and smoking in a multivariate model [88]. These results suggest that inflammation as evidence by higher CRP and elevated platelet-monocyte complexes is closely related to the extent of atherosclerosis and indicates that leukocyte activation has an import role in the pathogenesis of atherosclerosis in South Asians. This implies that deficiency or lack of endogenous molecules such as lipoxins that inhibit leukocyte activation could initiate atherosclerosis [53,86,87].

## Conclusions and therapeutic implications

**Figure 2:**
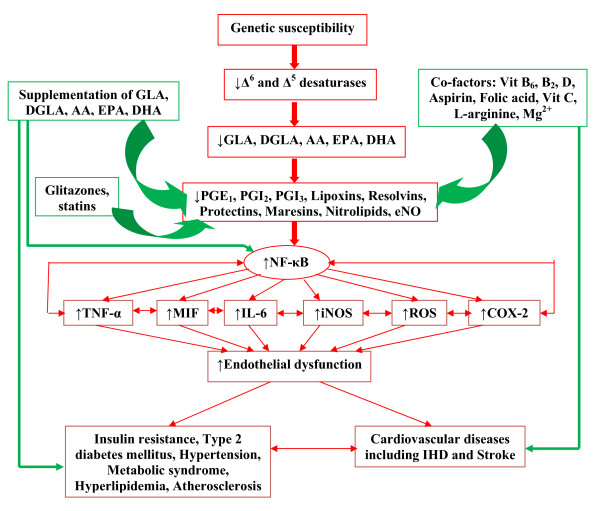
**Scheme showing the consequences of a defect in the activity of enzymes Δ^6 ^and Δ^5 ^desaturases and their relationship to the development of low-grade systemic inflammation and insulin resistance, type 2 diabetes mellitus, the metabolic syndrome, hypertension, atherosclerosis and IHD**. Supplementation of various PUFAs, and the co-factors that arenecessary for the adequate formation of PGE_1_, PGI_2_, PGI_3_, lipoxins, resolvins, protectins, maresins, nitrolipids and endothelial nitric oxide are expected to prevent, halt or even reverse the low-grade systemic inflammation and help in the prevention and management of insulin resistance, type 2 diabetes mellitus, the metabolic syndrome, hypertension, atherosclerosis and IHD as shown in the figure. Green arrows indicate enhancement in the formation of PGE_1_, PGI_2_, PGI_3_, lipoxins, resolvins, protectins, maresins, nitrolipids and endothelial nitric oxide and prevention, halt and/or reversal of low-grade systemic inflammation, insulin resistance, type 2 diabetes mellitus, the metabolic syndrome, hypertension, atherosclerosis and IHD. Red arrows indicate how a defect in the activity of enzymes Δ^6 ^and Δ^5 ^desaturases could lead to a decrease in the formation of PGE_1_, PGI_2_, PGI_3_, lipoxins, resolvins, protectins, maresins, nitrolipids and endothelial nitric oxide and initiation and progression of low-grade systemic inflammation, insulin resistance, type 2 diabetes mellitus, the metabolic syndrome, hypertension, atherosclerosis and IHD.

It is evident from the preceding discussion that one significant factor that could render South Asians highly susceptible to develop insulin resistance, type 2 diabetes mellitus, the metabolic syndrome, IHD and atherosclerosis could be a defect in the activity of Δ^6 ^and Δ^5 ^desaturases and consequent lower plasma and tissue concentrations of PUFAs such as GLA, DGLA, AA, EPA and DHA and reduced formation of anti-inflammatory products PGE_1_, PGI_2_, PGI_3_, lipoxins, resolvins, protectins, maresins and nitrolipids that inhibit leukocyte activation and enhance insulin action. This implies that bypassing block in the activities of Δ^6 ^and Δ^5 ^desaturases by supplementing GLA, DGLA, AA, EPA and DHA and co-factors that are essential for the metabolism of EFAs so that adequate amounts of PUFAs could be formed endogenously such as low-dose aspirin, folic acid, vitamin B_6_, B_2_, L-arginine (to decrease asymmetrical dimethyl arginine, ADMA, levels that are increased in patients with IHD, type 2 diabetes mellitus, the metabolic syndrome and hypertension; ADMA also interferes with eNO synthesis), vitamin C and Mg^2+ ^(magnesium is an essential for the normal activity of Δ^6 ^and Δ^5 ^enzymes) will not only improve the plasma and cell/tissue content of various PUFAs but also augment production of beneficial PGE_1_, PGI_2_, PGI_3_, lipoxins, resolvins, protectins, maresins, nitrolipids and endothelial nitric oxide (eNO) and thus, prevent and/or improve insulin resistance, hypertension, type 2 diabetes mellitus, the metabolic syndrome and IHD [5-7,13,14,16-21,23,24,26,27,53-62,83-87,89-92] (see Figure 2).

Furthermore, previously I and my colleagues showed that AA, EPA and DHA protect pancreatic β cells from chemical-induced cytotoxicity [89-92] and thus preserve their insulin producing capacity. These results are supported by the recent study wherein it was shown that mfat-1 transgenic mice which produce higher amounts of ω-3 PUFAs than the wild type showed increased insulin secretion stimulated by glucose, amino acids and glucagon-like peptide and when challenged with TNF-α, IL-6 and γ-interferon the transgenic islets completely resisted cytokine-induced cell death [93]. These results suggest that certain PUFAs and their products [94,95] have β cell cytoprotective actions and thus, help in the preservation of insulin secreting capacity. Similar to some PGs, lipoxins, resolvins, protectins, maresins and nitrolipids have been shown to have cytoprotective properties [96-98]

In view of the critical role of Δ^6 ^and Δ^5 ^desaturases in essential fatty acid metabolism, it is important to study their genetic polymorphism in South Asians and correlating these findings with the severity of insulin resistance, type 2 diabetes mellitus and atheroslcerosis in the carotid arteries, peripheral vascular tree, and coronary arteries. It was reported that there is a close correlation between polymorphisms of human Δ^6 ^and Δ^5 ^desaturase genes FADS1 FADS2 and fatty acid composition in serum phospholipids. Eighteen polymorphisms located in this gene cluster located at chromosome 11q12-11q13.1, a region repeatedly found to be linked with atopy and other complex diseases were genotyped in 727 adults [99-101]. Polymorphisms and statistically reconstructed haplotypes of FADS1 and the upstream region of FADS2 showed strongest associations with the level of the direct precursor of inflammatory eicosanoids, the ω-6 fatty acid AA, also strong associations with levels of the ω-6 fatty acids LA, GLA, C20: 2 ω-6, DGLA, C22: 4 ω-6 and of the ω-3 fatty acids ALA, EPA and C22:5 ω-3. Such studies of polymorphisms of human Δ^6 ^and Δ^5 ^desaturase genes in South Asians may give clues to predict and assess the progress of insulin resistance, atherosclerosis and IHD.

Peripheral leukocytes-platelet mixed cultures could be supplemented with AA, EPA and DHA and the formation of lipoxins, resolvins, protectins, maresins, nitrolipids and endothelial nitric oxide could be tested in South Asians to identify those who are unable to form adequate amounts of these lipid mediators and NO. Such subjects need supplementation of various PUFAs, aspirin, glitazones, statins and other co-factors even if they appear to be apparently normal to prevent the development of insulin resistance, atherosclerosis and IHD in future. Retesting such subjects after supplementation of PUFAs, aspirin, glitazones, statins and co-factors to see whether formation of lipoxins, resolvins, protectins, maresins, nitrolipids and eNO has been enhanced will be necessary to ascertain their response to the treatment.

Plasma, RBC, leukocyte and platelet content of various PUFAs, lipoxins, resolvins, protectins, maresins, nitrolipids, NO and other eicosanoids such as PGs, LTs, and TXs (thromboxanes) and activities of Δ^6 ^and Δ^5 ^desaturases could be measured both before and after treatment and used as markers to predict future development and prognostic markers of insulin resistance, the metabolic syndrome, atherosclerosis and IHD. Urinary levels of lipoxins, resolvins, protectins, maresins and nitrolipids and isoprostanes could also be measured and used as biochemical indices of response to treatment and more so in those with diabetic nephropathy.

Development of small molecules that could specifically up regulate the activities of Δ^6 ^and Δ^5 ^desaturases may be useful in the prevention and progression of insulin resistance, the metabolic syndrome, atherosclerosis and IHD. Statins and glitazones modulate the activities of Δ^6 ^and Δ^5 ^desaturases. Hence, development structural analogues of statins and glitazones that specifically target Δ^6 ^and Δ^5 ^desaturases could be attempted. Since Δ^6 ^and Δ^5 ^desaturases are present in many tissues, it is anticipated that drugs that enhance the activities of Δ^6 ^and Δ^5 ^desaturases are likely to have other beneficial actions such as in the treatment of hypertension, dyslipidemia, type 2 diabetes mellitus and Alzheimer's disease. Since PUFAs are naturally occurring endogenous substances, present in almost all tissues and are essential components of all mammalian cells, it is likely that overexpression of Δ^6 ^and Δ^5 ^desaturases is unlikely to have any significant side effects. This is evident from the fact that Eskimos consume large amounts of marine fish that are rich in ω-3 fatty acids EPA and DHA and are not known to suffer from any significant side effects. Similarly, when PUFAs have been administered to different types of patients for long periods of time (from few months to few years) it was noted that there were not any significant side effects. It is also proposed that a defect in the activities of enzymes Δ^6 ^and Δ^5 ^and desaturases and consequent low plasma and tissue levels of GLA, DGLA, AA, EPA and DHA and their products lipoxins, resolvins, resolvins, protectins, maresins and nitrolipids may play a significant role in the development of insulin resistance, atherosclerosis, the metabolic syndrome and IHD in Europeans and other populations.

## Competing interests

The authors declare that they have no competing interests.

## Authors' contributions

UND is the sole contributor to this work.
